# Barriers and enablers to switching from a solid to a liquid formulation of Parkinson’s medication: a theory-based mixed methods investigation

**DOI:** 10.1007/s11096-022-01446-z

**Published:** 2022-07-16

**Authors:** Bethany Atkins, Debi Bhattacharya, Caroline Smith, Sion Scott

**Affiliations:** School of Allied Health Professions, University of Leicester, Leicestershire, LE1 7RH UK

**Keywords:** Adherence, Behavioural science, Determinants, Medicines, Medicines optimisation, Movement disorders, Parkinson’s disease, Prescribing

## Abstract

**Background:**

Swallowing tablets/capsules can become difficult and dangerous for People with Parkinson’s (PwP) who develop oropharyngeal dysphagia. Switching to a liquid delays the need for progressing to last line patches/injections. However, liquids are rarely used therefore a change in prescribing practice is warranted but, as with any change in behaviour, may be met with resistance.

**Aim:**

To characterise PwPs and carers’ barriers and enablers (determinants) of switching from solid to liquid Parkinson’s medication formulations.

**Method:**

Underpinned by the Theoretical Domains Framework, focus groups with PwPs and carers were convened to identify determinants of switching, which were then used to develop a questionnaire distributed across the UK. Determinants were prioritised if ≥ 50% of respondents agreed/strongly agreed that they were important to their decision to switch to a liquid formulation. Percentage precisions were reported as 95% confidence intervals.

**Results:**

From three focus groups and 131 questionnaires responses, PwPs and carers prioritised nine determinants. Three enablers had almost unanimous agreement: liquids’ flexibility for incremental dosing (72% ± 8); decline in Parkinson’s control (72% ± 8); prescriber’s endorsement to switch (70% ± 8). The barriers: perception that tablets/capsules are easier to dose than liquids (72% ± 8); and prescriber’s opposition to switching (70% ± 8), attracted similarly high agreement.

**Conclusion:**

There is a desire to switch to liquids when Parkinson’s progresses and for their use beyond this to offer flexibility in dosing, a previously unrecognised indication for switching. The only notable resistance to switching may be addressed by innovations from the pharmaceutical industry to make liquids easier to measure.

**Supplementary Information:**

The online version contains supplementary material available at 10.1007/s11096-022-01446-z.

## Impact statements

- This is the first study applying behaviour change theory to explore the key barriers and enablers that require addressing when prescribers work with people with Parkinson’s and their carers to switch from solid to liquid medicine formulations.
- There is a desire to switch to liquids when Parkinson’s progresses, and for their use beyond this to offer flexibility in dosing, which is a previously unrecognised indication for switching.
- Through the application of a theoretical framework of behaviour change theory, a range of theory and evidence-based behaviour change techniques that may be selected from to support safe and acceptable formulation switching in Parkinson’s are reported.
- Prescribers and the pharmaceutical industry should be guided by the relevant barriers and enablers characterised, and the identified behaviour change techniques, to inform switching and the optimal characteristics of a liquid formulation and its method of administration.

## Introduction

Parkinson’s is a progressive neurodegenerative condition characterised by tremor, muscle rigidity and bradykinesia [1] caused by a loss of dopamine containing cells in the basal ganglia. More than 80% of People with Parkinson’s (PwP) develop oropharyngeal dysphagia (OD), which is difficulty swallowing [2]. Oropharyngeal dysphagia challenges the intake of solids such as foods and medicines. Some patients may respond by limiting their oral intake, which can lead to malnutrition and medication non-adherence [3]. Others may attempt to persevere, which risks solids intended for the stomach depositing in the lungs [4].

There are no disease modifying treatments for Parkinson’s, however available therapies are highly effective in managing symptoms [5]. Treatment efficacy relies on medication adherence and even small deviations from prescribing directions can result in exacerbation [6].

Solid oral medicine formulations such as tablets and capsules are the mainstay of Parkinson’s treatment [5]. People with Parkinson’s are highly motivated to control symptoms and are more likely to adhere to prescribed medication relative to the general population [7]. They have a strong preference for treatment to be tailored and they are motivated to assume a degree of freedom to self-optimise their therapy [8].However OD presents a significant barrier to adherence to solid formulations [7]. Liquid formulations are an alternative to solids and are usually more suitable for people with mild to moderate OD [9]. Liquids are easier and safer to swallow because the bolus enters the pharynx at a slower rate, allowing more time for the epiglottis to protect the trachea and vocal folds when a swallow is triggered [10]. Moreover, liquids may be more acceptable to people without OD in some circumstances. A 2018 study investigating preference for solid versus liquid formulations of alendronic acid in people without OD found that the liquid performed better in terms of taste, appearance and tolerability [11].

Switching from a solid to a liquid is a potential treatment option for PwP who develop OD [5]. However, as most medications to treat Parkinson’s are not available as liquid versions [12], current practice is to maintain solid oral formulations for as long as possible, sometimes by the unlicensed crushing and dispersing of tablets or capsules in water [13–15], before escalating to non-oral formulations such as patches and injections [5]. There is a need to expand the repertoire of Parkinson’s treatments by increasing the availability of liquid formulations to enable prescribers to respond when patients develop OD. Increased availability and prescribing of liquid formulations to treat Parkinson’s constitutes a significant change in practice. Prescribers should ensure that any barriers and enablers, or ‘determinants’, to a switch from PwPs’ and carers’ perspectives are addressed to ensure safety and acceptability to prevent adverse changes in the behaviour such as medication non-adherence [11]. For example, patients have reported perceiving the tablet formulations to be more effective than liquids, despite bioequivalence and matched efficacy [11]. This may therefore present a barrier to switching for PwPs that prescribers would need to address, who are principally concerned with symptom control [7]. Moreover, patients may be concerned about the risks of using liquid medicines such as aspiration pneumonia with less viscous formulations [16], challenges measuring and administering liquids and potential challenges to storage [11]. A framework from which to draw strategies to address barriers and enablers facilitates effective patient behaviour change [17]. Such a framework should be developed through engagement with the target audience of behaviour change, in this case, PwPs and carers.

The importance of applying theory when designing strategies to change behaviour is widely recognised [18]. There are numerous theories relevant to medication taking behaviour and selecting any one theory may not fully reflect the range of constructs relevant to the behaviour. This may be overcome by selecting multiple theories, however, they often have overlapping constructs which introduces redundancy. The Theoretical Domains Framework (TDF) overcomes this challenge as it is a synthesis of 33 behaviour change theories for understanding and developing strategies to change behaviour organised into 14 theoretical domains [19]. Identifying the important domains for the target behaviour provides the theoretical understanding required to change behaviour. The 14 TDF domains are linked to a taxonomy of Behaviour Change Techniques (BCTs), which are strategies to change behaviour [20].

### Aim

Using the TDF, this study aimed to identify the barriers and enablers to safely and acceptably switching from solid to liquid Parkinson’s medication from the perspectives of PwP and carers. It also aimed to characterise the relative importance and generalisability of barriers and enablers, and identify relevant BCTs to inform the switching process.

### Ethics approval

Ethics and governance approval was granted by the University of East Anglia Faculty of Medicine and Health Research Ethics Committee (Reference: 2019/20-138, date: 17/06/2020) to undertake a mixed methods study with People with Parkinson’s (PwP) and carers.

## Method

This mixed methods study comprised of focus groups (Phase 1) to identify the theoretical determinants of switching to a liquid formulation followed by a questionnaire (Phase 2) to determine the relative importance and generalisability of the identified determinants. The consolidated criteria for reporting qualitative research (COREQ) checklist was used to support reporting of the study (Supplementary material 1).

### Phase 1: focus groups

Focus groups were selected to explore and gain a rich understanding of the theoretical determents of switching to a liquid formulation borne out of discussion between people with diverse experiences.

#### Participant identification and recruitment

People with Parkinson’s and informal carers were recruited via an expression of interest survey distributed via a UK Parkinson’s research charity newsletter. People with and without experience of switching to a liquid were eligible. We purposively sampled participants to attend three one-hour focus groups: two with PwPs and one with carers, each comprising three to six participants (n = 9 to 18 participants in total). This homogeneity was in recognition that PwPs are typically highly self-sufficient in managing and administering their medications [21, 22] and to aid group interaction and dynamics.

Sampling was to facilitate an even mix of demographic characteristics, UK geographical regions and experience of switching. Sampled participants completed an online consent form and they were remunerated with a shopping voucher. We followed the principles for deciding saturation in theory-based qualitative studies outlined by Francis et al. [23] to generate an informative and transferable sample that reflected the similarities and differences in attitudes to switching.

#### Data collection

We developed a semi-structured topic guide (Supplementary material 2) comprising of open questions and follow-up prompts to structure the discussions around the 14 TDF domains [19]. Patient and Public Involvement members from the partner UK Parkinson’s research charity reviewed the topic guide and we implemented their suggestions.

BA facilitated the focus groups in July 2020 and SS assisted (took field notes and audio-recorded the discussions). Facilitators were available for 30 min after for discussions with participants and to address any questions. After one focus groups, a participant enquired about carer support groups. In response we developed a bespoke information pack in collaboration with the charity for them.

All members of the research team have extensive experience of conducting qualitative research and DB and SS have extensive experience in qualitative research underpinned by behaviour change theory.

#### Data analysis

An administrator transcribed verbatim focus group recordings which were anonymised and checked for accuracy by BA. A concurrent thematic analysis was undertaken in NVivo 12 (QSR International) after each focus group. Data were analysed in two stages [24]: (1) inductive thematic analysis to identify barriers and enablers to switching, and (2) a deductive framework analysis to organise identified barriers and enablers into TDF domains to identify the behavioural determinants of switching.

At stage 1, BA coded familiarised data inductively for barriers and enablers to switching [25]. Using an inductive approach initially ensured the codes and resultant themes identified were strongly linked to the data, rather than restricted to the pre-defined TDF domains [24].

PwP and carer transcripts were initially coded separately before being combined and grouped into categories. Codes and emerging themes were compared within transcripts and across the dataset to ensure that the analysis accurately reflected both the shared and disparate views between the two participant groups.

Guided by the TDF domain definitions [19], the codes from stage 1 were mapped to the relevant TDF domain(s) through consensus discussion between the research team (stage 2). We organised codes into barriers and enablers to switching to liquids within each TDF domain. This process allowed continuous comparison of TDF mapping, and any disagreements were resolved through discussions.

### Phase 2: questionnaire

Informed by the phase 1 data, an online questionnaire was developed to measure the importance and generalisability of the identified barriers and enablers to switching. This also enabled prioritisation of the most important barriers, enablers and thus TDF domains that require addressing when prescribers work with PwP and carers to switch from solid to liquid formulations of Parkinson’s medication.

#### Questionnaire development and distribution

We generated items based on the barriers and enablers to switching identified in phase 1 thus fulfilling the relevance and comprehensiveness criteria for content validity [26]. Item(s) were generated for each identified barrier and enabler within the relevant TDF domain to measure the extent to which they influenced the decision to switch. We used positively and negatively phrased statements to reduce the risk of acquiescent response bias, whereby participants respond to each statement in a similar way [27]. Response options were on a 5-point Likert scale from strongly disagree to strongly agree. Patient and Public Involvement members piloted the questionnaire to identify any difficulties interpreting items and formulating responses. The questionnaire was refined iteratively until no further adaptations were deemed necessary to fulfil the content validity criterion of comprehensibility [26].

As a newly developed questionnaire, no existing data are available to inform a response size justification. A conservative estimate based on a distribution across the responses of 60% to 40% for a sample size of 120 provides a 95% confidence interval (CI) of ± 9% or smaller around the estimates of agreement with each questionnaire item. This is a sufficient respondent size to ensure that there is no overlap in CIs between the proportion of respondents agreeing and disagreeing with an item.

People with Parkinson’s and informal carers were invited to complete the questionnaire via an advert placed in the UK Parkinson’s research charity newsletter. The eligibility criteria were the same as for Phase 1. People with and without experience of switching to a liquid were eligible. To characterise our respondent population, we asked participants to provide the following demographic information: age range, gender, region of the UK, whether they were a PwP or a carer and whether they had previous experience of switching.

The questionnaire was hosted on Microsoft® Forms. Responses were collected between November 2020 and January 2021. We did not collect identifiable information and we assumed consent through participant completion of the questionnaire.

#### Data analysis

Data for PwP and carers were analysed separately IBM SPSS Statistics (version 27); descriptive statistics were used to characterise the respondent population and report item responses. Barriers and enablers were prioritised if ≥ 50% of respondents indicated that they agreed/strongly agreed that they were important to their decision to switch from solid to liquid formulations of Parkinson’s medication. The Theory and Techniques Tool [28] was used to identify and report the Behaviour Change Techniques (BCTs) linked to the prioritised barriers and enablers.

## Results

### Phase 1: focus groups

#### Sample

From the 98 People with Parkinson’s (PwP) and 17 carers (n = 115 in total) who expressed an interest, 25 were invited to participate in the focus groups. Of these, one declined, three did not respond and four did not attend their scheduled focus group.

The three focus groups comprised three to six participants (n = 17 in total); two included PwPs (n = 12) and one included carers (n = 5).

The median (IQ) age range of participants was 65–74 (55–64, 65–74), 9 (53%) were women and 14 (82%) had no previous experience of switching from solid to liquid formulations of Parkinson’s medication. Participants were from England, Scotland, Wales and Northern Ireland.

#### Thematic analysis

Six themes were generated through the stage 1 inductive thematic analysis: (1) the process of change; (2) impact on lifestyle; (3) margin for error; (4) reflections on ability to administer; (5) position in treatment pathway and (6) curiosities. Themes were recurring after the second focus group [23].

#### The process of change

People with Parkinsons’ attachment to current medication was expressed as a barrier to switching to a liquid. This attachment was most attributed to their unease with change.

The problem is we’re old and don’t like change. (Person with Parkinson’s 2, focus group 2)

Carers acknowledged this barrier, expressing that a switch might result in PwPs being unfamiliar with their medicine in a different formulation, which may lead to a perceived loss of independence and control over their Parkinson’s management.

There seems to be a reluctance once someone [with Parkinson’s] is established on a medication to change to a different type of formulation… it always raises suspicion as to it might be a different medication or it might have a different effect. (Carer 4, focus group 3)

However, PwPs’ attachment to the status quo was not an insurmountable barrier. Reassurance from prescribers and access to information and resources were proposed strategies.

I know my mum would have talked with her [Parkinson’s nurse] about what she thought about it and valued what she said because she knew my mum as a person so much better than the consultant, which is no reflection on the consultants because they haven’t got the time […] whatever the consultant thought about it she would have gone and talked to her Parkinson’s nurse (Carer 2, focus group 3)

#### Impact on lifestyle

A key consideration for switching is that liquids are generally less portable and more challenging to store, making them less convenient than the tablets and capsules that PwPs are used to. Challenges associated with travelling with liquids, especially for people working or going on holiday, was a particular concern.

If you’re still working and on liquid you’re having to take it in and out of work or else you have to get prescribed two bottles of the medication so there are you know just the physical nature of having a liquid I think presents problems on a practical level (Person with Parkinson’s 3, focus group 1)

The aforementioned potential for loss of independence associated with liquids could also impact on lifestyle as liquids are practically more difficult to administer and therefore PwP may require support which is unavailable when travelling.

Carers were also concerned about the potential for loss of independence, fearing that the work associated with the increased complexity of liquid administration would need to be absorbed by them. However, if a liquid version of a Parkinson’s medication was more effective, they supported a switch.

If the [liquid] delivered the actual drug in your body better then all of these things that we’ve talked about… syringes and filling it and getting the right amount and everything would all be put to one side (Carer 2, focus group 3)

Some PwPs agreed that efficacy took priority, but others felt quality of life convenience were more important.

If you were at the stage were you were choking taking tablets I mean obviously a liquid form would be much easier to take and if you were at that stage perhaps you wouldn’t be so concerned about you know travelling abroad… (Person with Parkinson’s 3, focus group 1)

This led to discussions amongst PwPs regarding their desire to appear ‘normal’ and to hide their Parkinson’s symptoms. Medicines administration, especially in social settings, was a potential risk to maintaining a sense of normality. They indicated that administering a liquid from a bottle would be less discreet than popping a tablet. Carers also felt that this would be important.

I’m quite a sociable person I like to go out to dinner […] and go to concerts and things and I have sort of a hip flask with water in and I can just I just quietly take a tablet without many people noticing […] that’s very important to me actually to be a normal person as near normal as I can be (Person with Parkinson’s 7, focus group 2)

Some PwPs felt that any advantage afforded by liquid Parkinson’s medicines are obsolete if they’re concomitantly prescribed non-liquid formulations for other conditions. Others suggested that there was merit in having a range of options for different situations, for example addressing a dislike of taking tablets and capsules in the morning when tablets and capsules are most difficult to swallow.

Sometimes when I wake up in the morning I think oh I’ve got to swallow flipping pills first thing and that’s when I wouldn’t mind a liquid then […] I just hate it in the morning that’s my worst time (Person with Parkinson’s 2, focus group 1)

Further, PwPs hypothesised that liquids may permit greater flexibility for incremental dose changes, noting that this liquid property would be advantageous in terms of an ease of personalising dosing regimens that is not possible with tablets or capsules.

When I was first starting on one tablet I had to try and cut it in half 2 mg was the smallest they made and I only wanted the one [1 mg] and that was you know was not very practical but with a liquid you could do that easily (Person with Parkinson’s 6, focus group 2)

#### Margin for error

Potential benefits of liquids were balanced with a perceived increased risk of things going wrong. Liquids are seen as more difficult to correctly administer compared to tablets or capsules. This heightened sense of risk was primarily associated with liquid doses not being pre-measured unlike for tablets and capsules. Overdoses and underdoses were therefore considered more likely.

One thing to consider is the size of the dose I mean I imagine it to be diluted to be any size it would be much better I think to have relatively large doses 20 50ml something like that cos then there’s if you do if you’re slightly out on the dose it has less of an impact whereas if you try and measure a tiny couple of ml you could probably over dose or under dose (Person with Parkinson’s 6, focus group 2)

Cognitive symptoms of Parkinson’s such as memory loss are also perceived to make scheduling and monitoring adherence to liquid doses more difficult, which carries risk. PwPs and carers noted that tablets and capsules are pre-measured, easily scheduled, compatible with support aids such as multi-compartment compliance aids, and distinctive in terms of colour and shape. Conversely, these were not recognised features of liquids, and therefore they may be less compatible for people with cognitive impairment.

My mum […] has a dosette box with a certain amount of tablets for each time that she has to take it in the day if you had a Parkinson’s meds in a liquid form as cognitively she goes downhill it will be very difficult for her to be on her own and have to remember to take that (Carer 1, focus group 3)

#### Reflections on ability to administer

Challenges associated with measuring, taking and generally handling liquid formulations are further barriers to switching. Participants reflected on how the symptoms of Parkinson’s could make using liquids more difficult to take than tablets or capsules. Challenges with accurate dose measurement as well as the potential to spill or drop liquids were notable.

if you knock the bottle over you’ve lost all that medication you need (Person with Parkinson’s 2, focus group 2)

#### Position in treatment pathway

Whilst liquids were perceived on the whole to be more difficult to administer, they were perceived to be easier and less painful to swallow for people with oropharyngeal dysphagia (OD) relative to tablets or capsules.

For people who are having problems with swallowing I have no doubt the liquid form is better (Person with Parkinson’s 5, focus group 1)

The perception that liquids are more suited to or more acceptable in the ‘future’ prevailed. This was expressed by some participants as liquids being the domain of healthcare professionals and healthcare settings. Specifically, participants noted that liquids should only be administered in healthcare settings to offset their perceived risks.

#### Curiosities

PwPs and carers’ limited experience and knowledge of liquid medicines extends to their uncertainties regarding the properties of a liquid medicine. Notably, uncertaincies regarding how liquids are absorbed, digested and whether or not they could be safely diluted led to speculative discussions that liquids might wear off quicker, need to be taken more frequently and may potentially be less effective. PwPs and carers also expressed concerns about the taste and possible side effects of liquids.

if it works quicker is there a downside you know later on after you’ve taken the liquid or does that mean you have to take the liquid if it’s faster acting more often smaller amounts more often (Person with Parkinson’s 3, focus group 1)

However, the speculative properties of liquids were also enablers. A consistent example proposed was that liquids might have a faster onset of action, therefore better treating the ‘off’ periods of Parkinson’s.

A liquid […] gets to the target quicker and therefore it’s less of an off time so that you take the meds and you can see the effect quicker (Carer 2, focus group 3)

### Phase 2: questionnaire

A 23 item questionnaire was developed: 16 items were barriers; four were enablers; and three could be either a barrier or an enabler.

One hundred and forty-two questionnaire responses were received (n = 131 PwPs and 11 carers). Carer data are provided in Supplementary material 3 but excluded from the results and analysis as the number of responses did not reach sufficient sample size to achieve our maximum confidence interval threshold (see methods).

The median (IQ) respondent age range was 65–74 years (55–64, 65–74), of whom 72 (55%) were men and 128 (97.7%) administered their own medicine. Only six (4.6%) reported prior experience of switching.

Table 1 provides PwPs responses to questionnaire items organised into the relevant Theoretical Domains Framework (TDF) domains. Eleven items (five barriers and three enablers) spanning six TDF domains fulfilled the criterion for prioritisation. Of the five enablers, three had almost unanimous agreement: the flexibility offered by liquid medicines to allow for incremental dosage changes (72% ± 8), decline in Parkinson’s control (72% ± 8) and prescriber’s endorsement of a switch (70% ± 8). Of the four barriers, only the perception that tablets/capsules would be easier to correctly dose than measuring liquids (72% ± 8) and prescriber’s opposition to a switch (70% ± 8) attracted similarly high agreement.

**Table 1:** People with Parkinson’s responses to statements regarding potential barriers and enablers

Item	Theoretical domain Statement	Strongly disagree number (%)	Disagree number (%)	Neither agree nor disagree number (%)	Agree number (%)	Strongly agree number (%)
	*Beliefs about capabilities*					
1	I think I would find it more difficult to measure the right amount of liquid medicine^a^	9 (6.9)	44 (34.6)	22 (16.8)	48 (36.6)	8 (6.1)
	*Emotion*					
2	I would worry about getting the dose wrong with a liquid medicine^a^	15 (11.5)	50 (38.2)	29 (22.1)	31 (23.7)	6 (4.6)
3	The idea of using a liquid medicine makes me nervous^a^	20 (15.3)	57 (43.5)	35 (26.7)	18 (13.7)	1 (0.8)
	*Behavioural regulation*					
4	I would find it more difficult to check whether I’d taken the correct amount of liquid medicine^a^	14 (10.7)	33 (25.2)	24 (18.3)	49 (37.4)	11 (8.4)
	*Beliefs about consequences*					
5	I think a liquid medicine would take effect quicker than a tablet or capsule^b^	0	2 (1.5)	41 (31.3)	79 (60.3)	9 (6.9)
6	I don’t think a liquid medicine would be as effective at managing my Parkinson’s^a^	12 (9.2)	44 (34.6)	71 (54.2)	4 (3.1)	0
	*Environmental context and resources*					
7	A benefit of liquid medicine is it’s flexible for small changes to allow my dose to be personalised^b^	0	6 (4.6)	31 (23.7)	79 (60.3)	15 (11.5)
8	The storage of a liquid medicine would be less practical^a^	6 (4.6)	35 (26.7)	36 (27.5)	44 (33.6)	10 (7.6)
9	The administration of a liquid medicine would be less practical^a^	7 (5.3)	35 (26.7)	22 (16.8)	52 (39.7)	15 (11.5)
10	Pre-measured doses, such as tablets and capsules, are easier than trying to measure the right dose of a liquid medicine^a^	4 (3.1)	13 (9.9)	20 (15.3)	69 (52.7)	25 (19.1)
11	I would find tablets or capsules easier to swallow than a liquid medicine at the moment^a^	20 (15.3)	56 (42.7)	38 (29.0)	14 (10.7)	3 (2.3)
12	It’s important to me that a liquid medicine has a nice taste and texture^a/b^	1 (0.8)	15 (11.5)	46 (35.1)	55 (42.0)	14 (10.7)
13	There would be no point in having my Parkinson’s medicines as a liquid if I had other medicines that were tablets or capsules^a^	10 (7.6)	47 (35.9)	31 (23.7)	39 (29.8)	4 (3.0)
	*Skills*					
14	I would find it physically difficult to open and/or measure out the dose of a liquid medicine^a^	9 (6.9)	50 (38.2)	30 (22.9)	38 (29.0)	4 (3.1)
15	Consuming all of the dose of a liquid medicine would be difficult^a^	20 (15.3)	74 (56.5)	25 (19.1)	11 (8.4)	1 (0.8)
	*Memory, attention and decision processes*					
16	I think I would find remembering to take a liquid medicine more difficult^a^	22 (16.8)	55 (42.0)	26 (19.8)	24 (18.3)	4 (3.1)
	*Social influences*					
17	I would be self-conscious about taking a liquid medicine in public^a^	10 (7.6)	40 (30.5)	23 (17.6)	54 (41.2)	4 (3.1)
18	The opinion of my prescriber about the decision to switch to a liquid medicine is important to me^a/b^	3 (2.3)	7 (5.3)	29 (21.1)	79 (60.3)	13 (9.9)
19	The opinion of my family and/or friends about the decision to switch to a liquid medicine is important to me^a/b^	7 (5.3)	50 (38.2)	46 (35.1)	26 (19.8)	2 (1.5)
20	If I had the right support network to help me (e.g., family, friends and/or carers), I’d be more likely to switch to a liquid medicine^b^	2 (1.5)	38 (29.0)	59 (45.0)	28 (21.4)	4 (3.1)
	*Social, professional role and identity*					
21	Liquid medicines are best administered by healthcare professionals^a^	19 (14.5)	62 (47.3)	42 (32.1)	7 (5.3)	1 (0.8)
	*Intentions*					
22	I’d be reluctant for changes to be made to any medicines that I have been taking for a long time^a^	14 (10.7)	47 (35.9)	27 (20.6)	35 (26.7)	8 (6.1)
23	I’d be willing to switch to a liquid medicine if my Parkinson’s got worse^b^	0	3 (2.3)	34 (26.0)	70 (53.4)	24 (18.3)

Shaded items indicate that they met the prioritisation criterion of at least 50% of respondents reporting that they agreed or strongly agreed that the barrier or enabler was important to their decision to switch from solid to liquid formulations of Parkinson’s medication

^a,b^Whether the item is a barrier, enabler or both

**Fig. 1:**
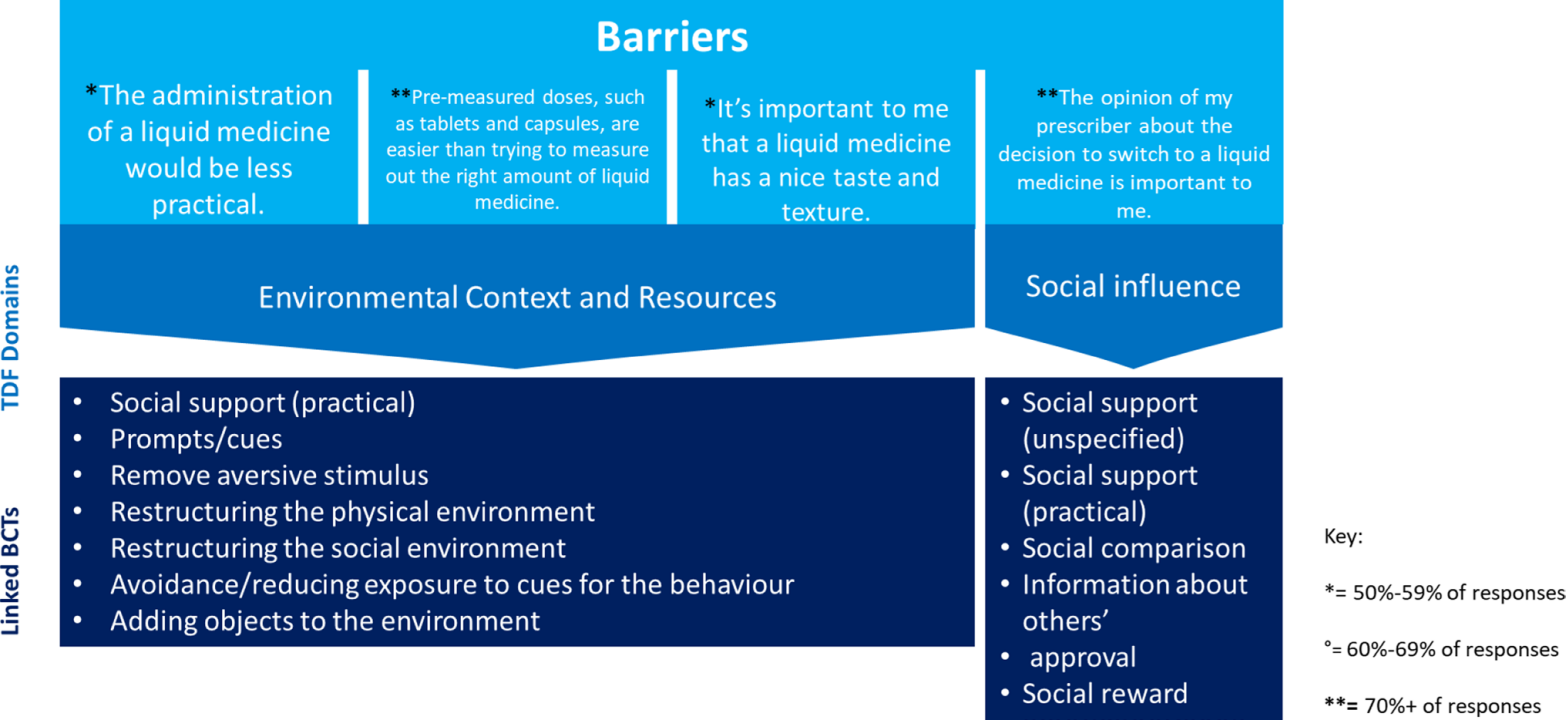
Prioritised barriers to switching from solid to liquid formulations of Parkinson’s medication and their associated Behaviour Change Techniques (BCTs)

**Fig. 2:**
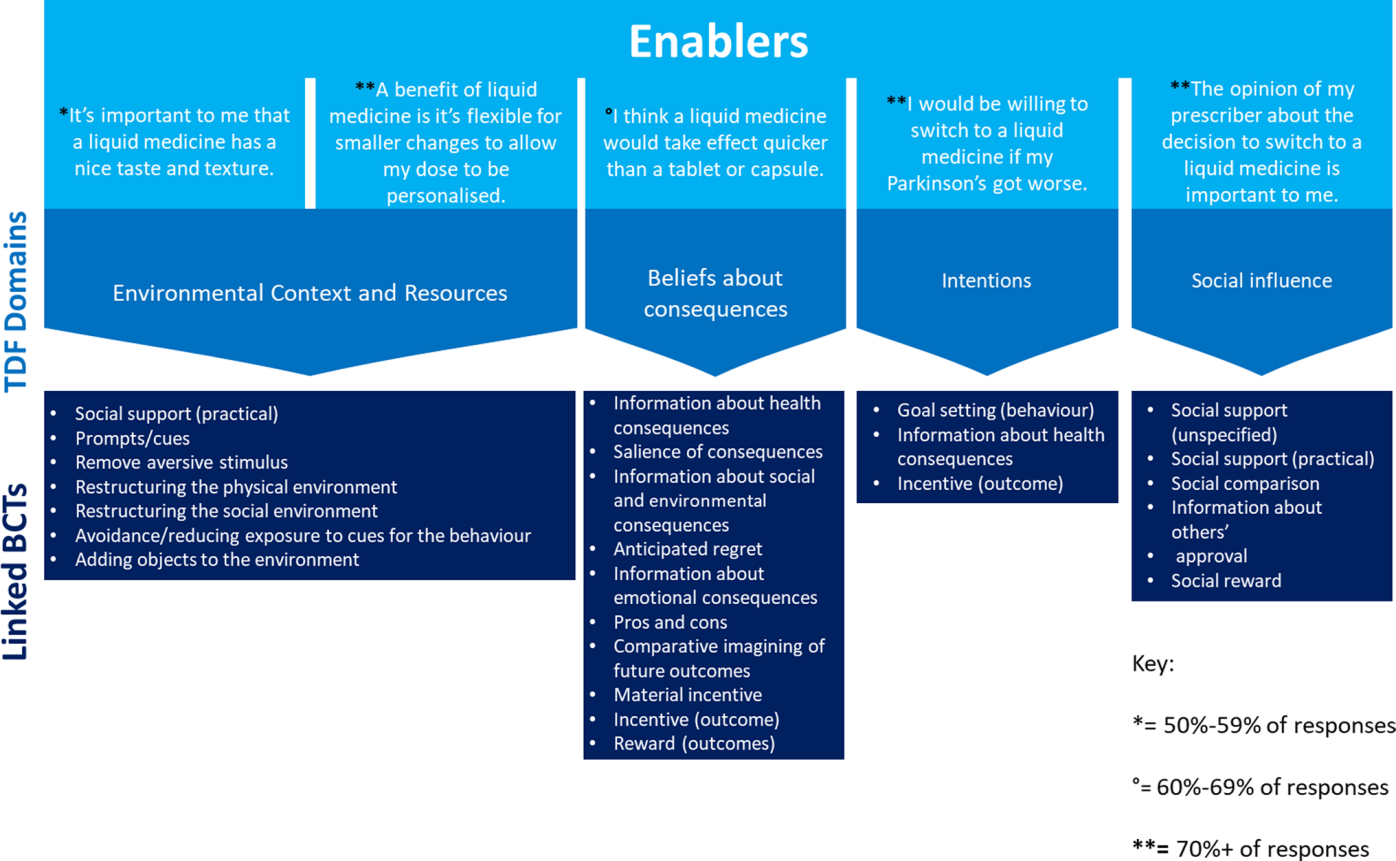
Prioritised enablers to switching to liquid versions of Parkinson’s medication

The five enablers were mapped to four TDF domains. Three barriers were mapped to the Environmental Context and Resources TDF domain and one to Social Influence. Figures 1 and 2 provide the barriers and enablers to switching from solid to liquid formulations of Parkinson’s medication, the corresponding TDF domains and the linked Behaviour Change Techniques.

## Discussion

People with Parkinson’s (PwP) and their carers expressed that liquid formulations have two roles in Parkinson’s treatment. The first is for PwPs who develop oropharyngeal dysphagia (OD); a switch to a liquid formulation is a necessary and reasonable adjustment to preserve medicines administration. The second is to enhance PwPs experience of taking their medicines by offering increased flexibility for incremental dose changes. This aligns with PwPs typically taking a proactive role in managing their medicine regimes and symptoms [21, 22]. The flexibility of liquids aligns with this desire for proactive management by offering PwPs increased control over their symptoms [29].

People with Parkinson’s’ desire for the flexibility offered by liquids needs to be balanced against practical considerations. While the data suggest a desire for liquids, the ‘Environmental Context and Resources’ domain requires addressing to alleviate the perceived practical challenges associated with administering and transporting liquids. Development of pre-measured and adaptable liquid formulations may provide a solution. A pre-measured and adaptable process of administration would provide flexibility regarding PwPs daily needs and fluctuating symptoms whilst also assimilating into their lifestyles. For example, pre-measured doses would allow PwPs to take their liquid medicine more easily whilst exercising, at work or social events. An increase in the availability and range of pre-measured doses would therefore work to address the barriers around the practicalities of administration and portability in that such methods would make liquids more amenable to PwPs’ individual lifestyles and symptoms.

People with Parkinson’s’ expressed in the focus groups that liquids are less practical to administer in comparison to tablet or capsules owing to the motor symptoms of Parkinson’s, which led to concerns that they would result in a loss of independence. However, this hypothesised barrier did not translate into reporting of PwPs having insufficient ‘Skills’ to use liquids. Similarly, while medication adherence is generally poor for older people [30], the hypothesised barrier was also contradicted by the questionnaire data. While focus group participants speculated that symptoms such as memory loss could make medication adherence more difficult, this did not lead to them expressing in the questionnaire that they would be less confident in their ability to remember to take liquids.

A recurring link throughout the themes is PwPs and carers perceptions that liquids present more scope for errors, such as over and under dosing. An admitted lack of understanding is often associated with risk and unreasonable fears [31]; in their study with PwPs, Kelly, et al*.* reported that a lack of knowledge would stop them from using liquid medicines [32]. The data in this study mirrors their findings [32]. It is not a lack of confidence in PwPs capabilities that is a barrier to switching, but the knowledge gap around the method and/or practicalities of using liquids that influences their attitudes towards them. Again, targeting the ‘Environmental Context and resources’ domain and promoting pre-measured doses would help to mitigate the issues of practicality and the perceived increase in risk.

Prescribing is based on a partnership between the patient who is the expert in their illness and the prescriber who is the disease and treatment expert [33]. Whilst PwPs gravitate towards the automatous end of the health decision-making continuum, they nonetheless indicate that the opinion of prescribers is an important influencer of switching. Whilst a prescribers’ favourable view of liquids is an enabler; their opposition is a barrier. Incorporating liquids into routine Parkinson’s treatment therefore requires practitioners to share the same enthusiasm for liquids expressed by PwPs.

Whilst this study represents a national sample of the UK; this may limit the international transferability of findings. The majority of participants having no previous experience of switching also means that the views of those with experience, and thus other potentially relevant barriers and enablers to switching, have not been sufficiently captured. The vast majority of PwP in this study sample reporting that they are independent in administering their medication may explain the low number of carer questionnaire responses. Recruiting via a newsletter likely introduced self-selection bias, with the participant population in this study being younger than the average population with Parkinson’s and sufficiently independent to self-enrol in a research study [34]. It is possible that a population of PwP with more advanced disease, and carers who are known to play a significant role in managing their medication [35], may have substantially different views that are not represented in this study.

## Conclusion

There is a desire to switch to liquids when Parkinson’s progresses and for their use beyond this to offer flexibility in dosing which is a previously unrecognised indication for switching. The only notable resistance to switching may be addressed by innovations from the pharmaceutical industry to make liquids easier to measure. The influencers of switching to a liquid version of a Parkinson’s medication that require addressing are mapped to 40 Behaviour Change Techniques. This provides an evidence and theory-based framework from which strategies may be selected to support a safe and acceptable switch. There is a desire for liquid medications to be further represented in the treatment pathway for Parkinson’s; prescribers and the pharmaceutical industry should be guided by the relevant barriers and enablers characterised to inform switching and the optimal characteristics of a liquid formulation and its method of administration.

## Supplementary Information

Below is the link to the electronic supplementary material.

Supplementary file1 (DOCX 24 kb)

Supplementary file2 (DOCX 34 kb)

Supplementary file1 (DOCX 39 kb)
