# Supplementary material for: Barriers and enablers to switching from a solid to a liquid formulation of Parkinson’s medication: a theory-based mixed methods investigation
Source: Int J Clin Pharm. 2022 Jul 16;44(4):1046–56. doi: 10.1007/s11096-022-01446-z (PMC9393141; doi:10.1007/s11096-022-01446-z)
Supplement: Supplementary file 1 — Supplementary file1 (DOCX 24 kb) [file 11096_2022_1446_MOESM1_ESM.docx]

| **Item No.** | **Topic** | **Guide Questions/Description** | **Response** |
| --- | --- | --- | --- |
| **Domains 1: Research team and reflexivity** | | | |
| Personal characteristics | | | |
| 1 | Interviewer/facilitator | Which author/s conducted the interview or focus group? | BA and SS. |
| 2 | Credentials | What were the researcher’s credentials? E.g. PhD, MD | SS’s credentials were ‘MPharm, PhD’, DB’s credentials were ‘BPharm, PhD’, BA’s credentials were ‘BA, MA’, CS’s credentials were ‘BSc’. |
| 3 | Occupation | What was their occupation at the time of the study? | SS was a UK registered pharmacist and Lecturer in Behavioural Medicine, DB was a UK registered pharmacist and Professor of Behavioural Medicine, BA was a Senior Research Associate who was also undertaking a PhD  CS was a UK registered speech and language therapist |
| 4 | Gender | Was the researcher male or female? | SS (male) BA (female) CS (female) DB (female) |
| 5 | Experience and training | What experience or training did the researcher have? | All members of the research team have extensive experience of conducting qualitative research. DB and SS have extensive experience in qualitative research underpinned by behaviour change theory specifically.  SS completed training in qualitative research methodology and the principles and practice of behaviour change research. |
| Relationship with participants | | | |
| 6 | Relationship established | Was a relationship established prior to study commencement? | There were no established relationships between the researchers and the focus group participants. A relationship was established between the researchers and gatekeepers (UK Parkinson’s charity) for each group of participants for the purposes of recruitment. |
| 7 | Participant knowledge of the interviewer | What did the participants know about the researcher? e.g. personal goals, reasons for doing the research | Participants were informed of the research aims and objectives. |
| 8 | Interviewer characteristics | What characteristics were reported about the interviewer/facilitator? e.g. Bias, assumptions, reasons and interests in the research topic | Participants were informed that SS was a pharmacist and that the research was part funded by a pharmaceutical company. |
| **Domain 2: Study design** | | | |
| Theoretical framework | | | |
| 9 | Methodological orientation and Theory | What methodological orientation was stated to underpin the study? e.g. grounded theory, discourse analysis, ethnography, phenomenology, content analysis | Thematic analysis as described by Braun and Clark (2006) underpinned by the Theoretical Domains Framework. |
| Participant selection | | | |
| 10 | Sampling | How were participants selected? e.g. purposive, convenience, consecutive, snowball | Participants were purposively sampled to maximise variation in demographic characteristics, location in the UK and experience of switching. |
| 11 | Method of approach | How were participants approached? e.g. face-to-face, telephone, mail, email | All participants were invited via email from BA. |
| 12 | Sample size | How many participants were in the study? | 17 participants. |
| 13 | Non-participation | How many people refused to participate or dropped out? Reasons? | All but one participant who was purposively sampled agreed to participate in the focus groups. The person who declined did not give a reason. 1 participant dropped out due to a clash with other commitments and 3 participants didn’t turn up to their scheduled focus group. |
| Setting | | | |
| 14 | Setting of data collection | Where was the data collected? e.g. home, clinic, workplace | All focus groups were convened online via Microsoft Teams. |
| 15 | Presence of non- participants | Was anyone else present besides the participants and researchers? | No. |
| 16 | Description of sample | What are the important characteristics of the sample? e.g. demographic data, date | Refer to table 1 for demographic data and data were collected between July and September 2020. |
| Data collection | | | |
| 17 | Interview guide | Were questions, prompts, guides provided by the authors? Was it pilot tested? | A semi-structured topic guide was designed to illicit participants’ views regarding switching. Probes to explore the 14 TDF domains were also included and used where necessary.  Refer to Appendix 2 for the full topic guide. |
| 18 | Repeat interviews | Were repeat interviews carried out? If yes, how many? | No. |
| 19 | Audio/visual recording | Did the research use audio or visual recording to collect the data? | Focus groups discussions were audio recorded. |
| 20 | Field notes | Were field notes made during and/or after the interview or focus group? | Field notes were made during the focus groups. |
| 21 | Duration | What was the duration of the interviews or focus group? | Focus groups lasted approximately 60 minutes. |
| 22 | Data saturation | Was data saturation discussed? | No. |
| 23 | Transcripts returned | Were transcripts returned to participants for comment and/or correction | No. |
| **Domain 3: Analysis and findings** | | | |
| Data analysis | | | |
| 24 | Number of data coders | How many data coders coded the data? | BA inductively coded for the thematic analysis which was checked by SS and DB (qualitative research experts). BA, SS and DB mapped codes to the TDF. |
| 25 | Description of the coding tree | Did authors provide a description of the coding tree? | The TDF was used as a basis for the coding tree. |
| 26 | Derivation of themes | Were themes identified in advance or derived from the data? | Inductive and deductive approaches were utilised. The phase 1 thematic analysis involved inductive coding of data and thus no pre-determined themes were applied. For the phase 2 mapping to the TDF, the pre-defined domains were deductively applied to the phase 1 data.. |
| 27 | Software | What software, if applicable, was used to manage the data? | Data were managed using NVivo 11. |
| 28 | Participant checking | Did participants provide feedback on the findings? | No. |
| Reporting | | | |
| 29 | Quotations presented | Were participant quotations presented to illustrate the themes/findings? Was each quotation identified? e.g. participant number | Quotations are provided to contextualise novel concepts and participant numbers are provided. |
| 30 | Data and findings consistent | Was there consistency between the data presented and the findings? | Data including quotations are provided in a manner consistent with the findings. |
| 31 | Clarity of major themes | Were major themes clearly presented in the findings? | The major themes are presented and explained in the results section. |
| 32 | Clarity of minor themes | Is there a description of diverse cases or discussion of minor themes? | Shared and disparate views between participants are reported and explained in the results and discussed in the discussion.  The TDF domains which were mapped onto the major themes and the constituent inductive codes are provided in Figures 1 and 2. |
