# Supplementary material for: Barriers and enablers to switching from a solid to a liquid formulation of Parkinson’s medication: a theory-based mixed methods investigation
Source: Int J Clin Pharm. 2022 Jul 16;44(4):1046–56. doi: 10.1007/s11096-022-01446-z (PMC9393141; doi:10.1007/s11096-022-01446-z)
Supplement: Supplementary file 2 — Supplementary file2 (DOCX 34 kb) [file 11096_2022_1446_MOESM2_ESM.docx]

Topic guide

| Prior to focus group | 1. Introduce self; name and role and introduce assistant moderator; name and role 2. I’d really like to thank you for giving up your time to help with our research 3. Today we are looking for you to discuss some of your thoughts about switching Parkinson’s medications from tablets or capsules to a liquid version of the same medicine 4. There are no right or wrong answers, we are interested in your thoughts and opinions so just be yourself and speak as honestly as possible. 5. As mentioned in the information leaflet we will be recording this session. 6. Anything that is said within the session will be treated confidentially, your responses will be stored in an anonymous format, and so your names will not appear in any report. 7. And we also ask that each of you treat what is said within this session with the same confidentiality, and don’t share other people’s responses. Though, of course, you are free to share your own experience. 8. The session should take no more than one hour 9. For the purposes of the recording, please try not to talk over somebody as the recording may not capture what you are both saying 10. Finally, please interact with each other and bounce your ideas and thoughts around the group. We are only here to guide the discussion and if we end up saying nothing for the next hour – that’s fine! 11. Are there any questions before we begin? | Check all participants have completed the online consent forms  Camtasia – open and ready to start  Dictaphone  Notebook |
| --- | --- | --- |
| Switch on the recording devices (Camtasia and dictaphone) | - Confirm current status of each participant   - Can you please confirm your name for the recording? |  |

| **Question** | **Notes** |
| --- | --- |
| **Scene setting**  For some people with Parkinson’s, moving away from tablets or capsules to a liquid version is the preferred way forward. We know from previous work with other conditions that the patient’s attitudes and experiences of tablets and capsules are different to liquids. So even though it is the same medicine, because it is a liquid instead of a tablet or capsule, the person feels differently about it. We found in previous research that people thought the liquid was better in some ways and worse in others. This means that healthcare professionals need to take these thoughts into account when considering switching from tablets or capsules to a liquid medicine. So today we want you to tell us what healthcare professionals need to consider when thinking about switching medicines for managing Parkinson’s.   1. What are your initial thoughts about switching from a tablet or capsule to a liquid form of the same medicine? |  |
| We’ve talked about quite a few things. Something we haven’t talked about are the practicalities of switching.   1. Can you think of any practical things with switching to a liquid medicine; good or bad? |  |
| 1. Is there anything not under your control that might affect you switching to liquid? |  |
| 1. What is your gut feeling about switching to a liquid?  - Why? |  |
| 1. Can you think of any disadvantages? |  |
| 1. What do you think the benefits would be? |  |
