# Supplementary material for: Barriers and enablers to switching from a solid to a liquid formulation of Parkinson’s medication: a theory-based mixed methods investigation
Source: Int J Clin Pharm. 2022 Jul 16;44(4):1046–56. doi: 10.1007/s11096-022-01446-z (PMC9393141; doi:10.1007/s11096-022-01446-z)
Supplement: Supplementary file 3 — Supplementary file1 (DOCX 39 kb) [file 11096_2022_1446_MOESM3_ESM.docx]

| Theoretical Domain  Statement | Strongly disagree Number | Disagree Number | Neither agree nor disagree Number | Agree Number | Strongly agree Number |
| --- | --- | --- | --- | --- | --- |
| Beliefs about capabilities | | | | | |
| I think I would find it more difficult to measure the right amount of liquid medicine. | 3 | 1 | 0 | 7 | 0 |
| The person I care for would find tablets or capsules easier to swallow than a liquid medicine at the moment. | 0 | 5 | 5 | 1 | 0 |
| Emotion | | | | | |
| I would worry about getting the dose wrong with a liquid medicine. | 3 | 2 | 1 | 4 | 1 |
| The idea of using a liquid medicine makes me nervous. | 2 | 3 | 4 | 2 | 0 |
| Behavioural regulation | | | | | |
| I would find it more difficult to check whether the person I care had taken the correct amount of liquid medicine. | 2 | 1 | 1 | 6 | 1 |
| Beliefs about consequences | | | | | |
| I think a liquid medicine would take effect quicker than a tablet or capsule. | 0 | 0 | 5 | 4 | 2 |
| I don’t think a liquid medicine would be as effective at managing the Parkinson’s for the person I care. | 1 | 1 | 6 | 2 | 1 |
| A benefit of liquid medicine is it’s flexible for small changes to allow the dose to be personalised for the person I care for. | 0 | 0 | 6 | 4 | 1 |
| Environmental context and resources | | | | | |
| The storage of a liquid medicine would be less practical. | 2 | 2 | 4 | 3 | 0 |
| The administration of a liquid medicine would be less practical. | 1 | 1 | 3 | 4 | 2 |
| Pre-measured doses, such as tablets and capsules, are easier than trying to measure the right dose of a liquid medicine. | 1 | 2 | 2 | 3 | 3 |
| It’s important to the person I care for that a liquid medicine has a nice taste and texture. | 0 | 0 | 3 | 5 | 3 |
| There would be no point in having Parkinson’s medicines as a liquid if the person I care for had other medicines that were tablets or capsules. | 0 | 1 | 5 | 2 | 3 |
| Skills | | | | | |
| The person I care for would find it physically difficult to open and/or measure out the dose of a liquid medicine. | 0 | 1 | 3 | 4 | 3 |
| The person I care for would find it difficult to consume all of the dose of a liquid medicine. | 2 | 2 | 4 | 2 | 1 |
| Memory, attention and decision processes | | | | | |
| I think I would find remembering to give the liquid medicine to the person I care for more difficult. | 2 | 1 | 6 | 2 | 0 |
| Social influences |  |  |  |  |  |
| The person I care for would be self-conscious about taking a liquid medicine in public. | 1 | 3 | 3 | 2 | 2 |
| The opinion of the person I care for about the decision to switch to a liquid medicine would be important. | 0 | 1 | 2 | 2 | 6 |
| The opinion of the prescriber about the decision to switch to a liquid medicine would be important. | 1 | 2 | 3 | 3 | 2 |
| The opinion of the family and/or friends of the person I care for about the decision to switch to a liquid medicine is important to me. | 0 | 4 | 5 | 1 | 1 |
| If the person I care for had the right support network to help me (e.g., family, friends and/or carers), I’d be more willing for them to switch to a liquid medicine. | 0 | 2 | 5 | 2 | 2 |
| Social, professional role and identity |  |  |  |  |  |
| Liquid medicines are best administered by healthcare professionals. | 1 | 5 | 3 | 2 | 0 |
| Intentions |  |  |  |  |  |
| I’d be reluctant for changes to be made to any medicines the person I care for had been taking for a long time. | 0 | 3 | 3 | 4 | 1 |
| I’d be willing to switch to the person I care for to a liquid medicine if their Parkinson’s got worse. | 1 | 1 | 4 | 4 | 1 |
